# Coral larvae are poor swimmers and require fine-scale reef structure to settle

**DOI:** 10.1038/s41598-017-02402-y

**Published:** 2017-05-22

**Authors:** Tom Hata, Joshua S. Madin, Vivian R. Cumbo, Mark Denny, Joanna Figueiredo, Saki Harii, Christopher J. Thomas, Andrew H. Baird

**Affiliations:** 1 Hopkins Marine Station, Stanford University, 120 Ocean View Blvd, Pacific Grove, CA 93950-3094 USA; 2 Department of Biological Sciences, Macquarie University, Sydney, New South Wales, 2109 Australia; 3 ARC Centre of Excellence for Coral Reefs Studies, James Cook University, Townsville, Queensland 4811 Australia; 4 Halmos College of Natural Sciences and Oceanography, Nova Southeastern University, 8000 N Ocean Drive, Dania Beach, FL 33004 USA; 5 Sesoko Station, Tropical Biosphere Research Center, University of the Ryukyus, Okinawa, Japan; 6 Université catholique de Louvain, Institute of Mechanics, Materials and Civil Engineering, Avenue G. Lemaître 4-6, B-1348 Louvain-la-Neuve, Belgium

## Abstract

Reef coral assemblages are highly dynamic and subject to repeated disturbances, which are predicted to increase in response to climate change. Consequently there is an urgent need to improve our understanding of the mechanisms underlying different recovery scenarios. Recent work has demonstrated that reef structural complexity can facilitate coral recovery, but the mechanism remains unclear. Similarly, experiments suggest that coral larvae can distinguish between the water from healthy and degraded reefs, however, whether or not they can use these cues to navigate to healthy reefs is an open question. Here, we use a meta-analytic approach to document that coral larval swimming speeds are orders of magnitude lower than measurements of water flow both on and off reefs. Therefore, the ability of coral larvae to navigate to reefs while in the open-ocean, or to settlement sites while on reefs is extremely limited. We then show experimentally that turbulence generated by fine scale structure is required to deliver larvae to the substratum even in conditions mimicking calm back-reef flow environments. We conclude that structural complexity at a number of scales assists coral recovery by facilitating both the delivery of coral larvae to the substratum and settlement.

## Introduction

Reef corals have evolved in a highly dynamic environment repeatedly subject to many types of disturbances; in particular storms and floods^1^ and, more recently, coral bleaching and mortality caused by global warming^2^. The scale and severity of many of these disturbances are predicted to increase in response to climate change^3^, and consequently there is an urgent need to improve our understanding of the mechanisms underlying reef recovery. For example, while reef structural complexity is often associated with increased rates of recovery, the precise mechanism is unknown^4^.

The recovery of reef coral assemblages from catastrophic disturbance is generally dependent on larval replenishment from other reefs^5, 6^ (but see ref. 7). Therefore, any process that increases larval supply should also assist recovery. For example, reefs with high levels of connectivity should recover more quickly than reefs isolated by distance or currents from sources of larvae^8, 9^. Similarly, reefs that are more effective at capturing larvae from the plankton should recover more quickly than other reefs^10^. Recovery is also dependent on successful settlement and recruitment. For example, the role of reef micro-structure, such as the crevasses excavated by echinoderms while grazing^11, 12^, in providing a refuge from predation^13–15^ and thereby enhancing post-settlement survivorship is well establish. In contrast, very little is known about the process of settlement on reefs. Under controlled laboratory conditions, numerous factors can influence whether or not a coral larva settles, including chemical cues and phototaxis^16, 17^. However, there is a paucity of direct observations of coral larval settlement in complex topographical and hydrodynamic environments^18, 19^.

The entrapment of larvae by reefs, their interaction with complex reef structure, and settlement are all likely to be influenced by larval swimming speeds and sensory capacity. An important historical theme in marine ecology has been the tendency to underestimate the capacity of larvae to influence their fate. Marine larvae were initially considered to be passive particles with little capacity to sense or respond to their environment^20–22^. These ideas led to the paradigm of massive export of larvae from the reef of origin followed by dispersal over a large spatial scale^23, 24^. However, subsequent research demonstrated that the larvae of many marine taxa can respond to a diverse array of environmental and chemical cues that give them some capacity to influence their fate^18, 25^. For example, crustacean larvae are able to remain in estuaries by performing tidally-synchronized vertical migrations^26^, and reef fish larvae can smell nearby reefs^27^ and swim towards them for sustained periods^28^. Similarly, patterns of dispersal among coral species are influenced by aspects of their biology, such as rates of larval development^29, 30^ and larval response to settlement cues^31, 32^. Nonetheless, the capacity of marine larvae to influence patterns of dispersal and settlement through swimming behavior is likely to be limited because the larvae of many taxa, in particular scleractinian corals, are very poor swimmers^20^. For example, even if coral larvae can distinguish between waters from healthy and degraded reefs^33^, it is highly unlikely they will be able to navigate to healthy reefs if their swimming speeds are less than currents in the open ocean.

Here, we first compare data from the literature on coral larval swimming speeds with empirical measurements of water flow both on and off reefs to explore the capacity of swimming ability to influence dispersal and settlement. We then test the ability of coral larvae to settle in a flume using current speeds that mimic the flow regime on reefs and explore the role of micro-structure in affecting settlement.

## Methods

### Larvae swimming speeds

We included all data on coral larval swimming speeds based on our knowledge of the literature, because most of these data are inaccessible to current search engines, such as the work of Japanese scientists in Palau in the 1930s and 1940s. Swimming speeds were measured as distance covered per unit time in all studies. The only data we filtered was from Harrigan^34^, where swimming speeds for only the first 7 days were used to allow direct comparisons with the other studies. The only data excluded was Hodgson^35^, because we do not accept that it is possible to identify coral larvae collected in plankton tows to species. Our meta-analysis included 9 studies representing over 450 measurements of individual larva from eight coral species (Table 1). Using R^36^, an ANCOVA was run on the log-transformed swimming speeds to test for an effect of larval size and swimming direction (horizontal, upwards and downwards) on swimming speeds. Larval size did not have a significant effect on swimming speed and removing it greatly improved the model based on Akaike’s Information Criterion. Differences among swimming directions in the final model were assessed using Tukey Honestly Significant Differences (*TukeyHSD*).

**Table 1:** Swimming speeds (in mm s^−1^) for hermatypic scleractinian coral larvae.

Species	Direction	Min	Max	Mean	SE	*n*	Length (mm)	Reference
*Heliofungia actiniformis*	Horizontal	1.15	1.90	1.57	0.09	8	0.50	^46^14
*Pocillopora damicornis*	Horizontal	1.67	1.88	1.78^	n/a	n/a	1.00	^47^15
*Pocillopora damicornis*	Horizontal	0.08	3.09	2.01*	0.07	82	1.18	^34^16
*Coelastrea aspera*	Horizontal	2.00	3.45	2.73	n/a	n/a	0.47	^48^17
*Heliofungia actiniformis*	Up	0.90	2.65	1.66	0.09	18	0.50	^46^14
*Agaricia tenuifolia*	Up	1.04	3.16	2.10	0.20	28	n/a	^49^18
*Galaxea horrescens*	Up	1.32	3.33	2.41	0.15	20	2.30	^50^19
*Pocillopora damicornis*	Up	1.61	4.50	2.79	0.11	30	2.00	^51^20
*Porites astreoides*	Up	1.26	4.34	2.80	0.20	59	0.75	^49^18
*Isopora bruggemanni*	Up	1.10	4.55	2.86	0.24	20	2.50	^52^21
*Seriatopora hystrix*	Up	n/a	n/a	3.33	n/a	n/a	1.50	^53^22
*Heliofungia actiniformis*	Down	1.97	3.80	2.76	0.17	9	0.50	^46^14
*Isopora bruggemanni*	Down	2.56	5.56	3.55	0.18	20	2.50	^52^21
*Agaricia tenuifolia*	Down	2.01	5.19	3.60	0.30	28	n/a	^49^18
*Galaxea horrescens*	Down	3.03	5.21	3.86	0.13	20	2.30	^50^19
*Porites astreoides*	Down	2.76	5.84	4.30	0.30	59	0.75	^49^18
*Seriatopora hystrix*	Down	n/a	n/a	4.44	n/a	n/a	1.50	^53^22
*Pocillopora damicornis*	Down	3.68	6.49	4.79	0.13	30	2.00	^51^20

*n* is number of larvae; SE is standard error; ^ is the mean calculated as average of maximum (max) and minimum (min) value; * is the mean calculated from larvae aged 2 to 7 days old; n/a is not available.

### Current speeds

Current speeds during the two weeks following coral mass spawning in six successive years at three locations in the Great Barrier Reef were obtained from the Great Barrier Reef Ocean Observing System^37^. The mooring sites were located in the vicinity of coral reefs at depths of 45-51 m (Heron Island North, 151.987°E, 23.380°S), 50–62 m (Heron Island South, 151.955°E, 23.513°S), and 31–37 m (Lizard Island Shelf, 145.641°E, 14.702°S). The current data were obtained from Acoustic Doppler Current Profilers. The water depth at the mooring sites was between 30 m and 60 m and the devices recorded water velocities at depth layers starting from 1–2 m above this level to the water surface. The magnitudes of horizontal and vertical currents were calculated at each depth layer, and these were averaged over the layers in the top 20 m of the water column to give a depth-average of horizontal and vertical current speeds.

### Measuring water motion on the reef

Water motion over the reef was measured on the fringing reef of Lizard Island, Australia, between South and Palfrey Islands (14.700°S, 145.449°E), known as Trimodal Reef. An acoustic Doppler velocimeter (ADV) was deployed from November 16 to 24 2013, recording samples at 8 Hz in a burst of 2,048 samples once every 20 min. Data were summarized by recording the mean velocity components for each burst in the toward-shore (u), along-shore (v) and vertical (w) directions for each burst. The spectra of individual bursts were examined to determine the dominant period of oscillation. Water velocities were measured using particle image velocimetry (PIV)^38^. A vertical plane of water parallel to water motion was illuminated by a laser sheet (300 mW, 532 nm). Waterborne particles within the laser sheet were filmed at 30 fps using a digital video camera in an underwater housing with a 532 nm band pass filter. Both laser and camera were attached to an aluminum tripod frame 40 × 40 × 30 cm (L × W × H) in size, and a black felt curtain was extended ~60 cm behind the laser sheet to reduce background light. Eight sites were chosen along a transect perpendicular to the reef crest and in line with the ADV (±2 m to either side along-shore), from the crest to 50 m towards shore. At each site, 2 min of video were recorded in an 8 × 5 cm (W × H) field of view (FOV) over a relatively flat area of the site, approximately central to the shoreward and seaward edges of the raised feature. The FOV was directly above the substrate, including the upper 0.5 to 1 cm of the substrate. Footage from two sites (located 1.0 m and 3.2 m behind the reef crest) were chosen for subsequent PIV analysis.

Video footage was stabilized using Deshaker software^39^ package in VirtualDub^40^ and 30 s of each two-min clip was then analyzed using PIVlab^41^ in Matlab (Mathworks). The velocity measurements (u, w) of each site were recorded as a single vertical velocity profile located centrally in the FOV for each frame, starting approximately 1 mm from the substrate and increasing in 1.3 mm height increments. For each site, mean toward-shore velocity (u) as a function of distance from substrate (h) was calculated across all processed frames. The velocity gradient was assumed to be linear between the substrate (where u = 0) and the closest velocity measurement to the substrate, a conservative estimate.

### Assessing settlement behavior of *Isopora cuneata* larvae

The study organism, *Isopora cuneata* (Family Acroporidae), a brooding coral, was chosen because of its high abundance on Trimodal Reef and the fast swimming speeds of congeners (Table 1). Branches of *I. cuneata* colonies were collected in the field and were placed in an outdoor flow-through seawater tank. Flow was suspended overnight and larvae were collected in the morning by pipette and kept in 0.2 μm filtered seawater (FSW) until used in the experiments.

**Figure 1:**
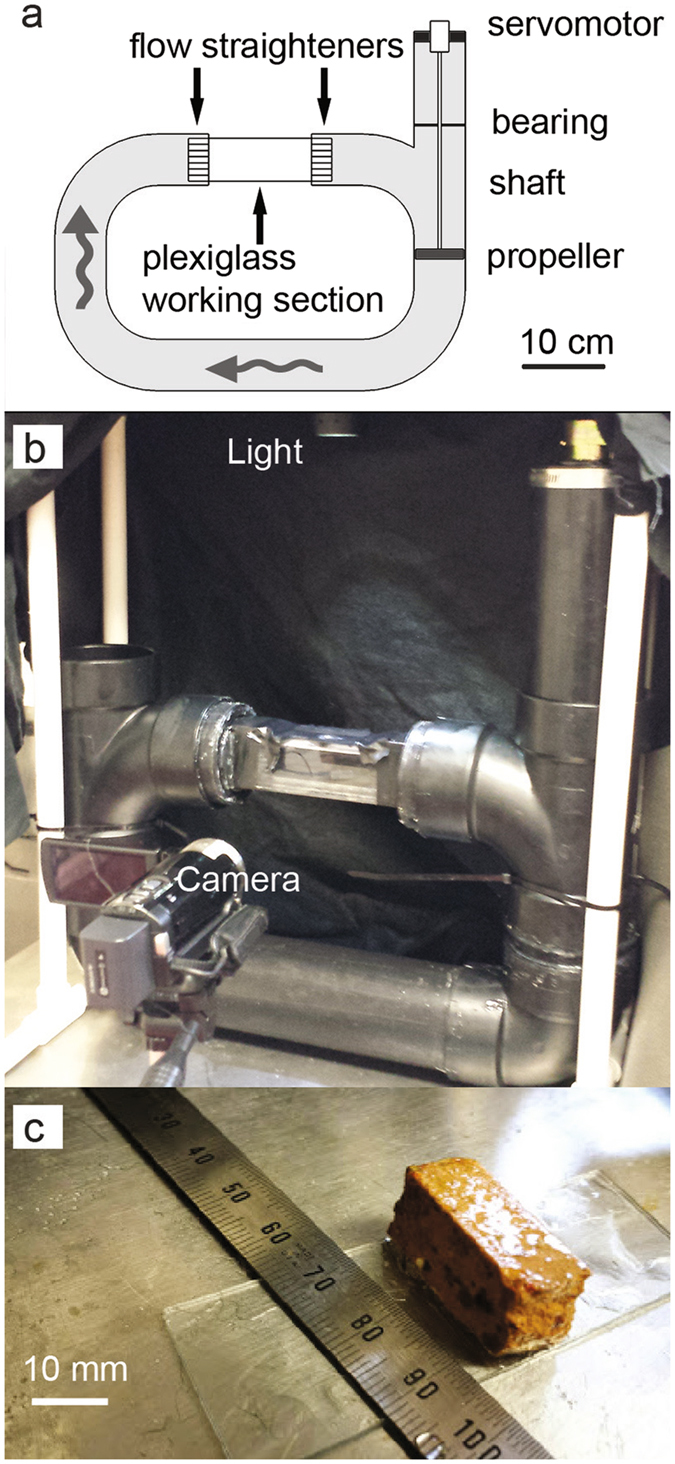
The oscillating flume tank for coral larval swimming tests, which was designed to replicate water motion from the reef (see Methods for a full description). (**a)** Schematic of the oscillating flume tank. (**b**) Picture of flume set-up with light and high-speed camera. (**c**) Picture of a block treatment settlement tile (1 × 2.5 × 1 cm, L × W × H) mounted on a glass slide.

To assess larval settlement behavior in flow conditions similar to those found on coral reefs, *I. cuneata* larvae were placed in a recirculating flume capable of generating oscillating water motion (Fig. 1a and b). The oscillating flume was composed of acrylonitrile-butadiene-styrene pipe (7.6 cm outer diameter; 45 × 30 cm, L × H) with a clear, rectangular plexiglass working section (15 × 2.8 × 5 cm inner L × W × H). Flow recirculated in a closed vertical loop, driven by a propeller located in the vertical arm of the flume downstream of the working section. The propeller was attached to a servomotor. Rotation rate of the servomotor was controlled by an amplified analog voltage signal output by a custom Matlab script and transduced by a data acquisition card (National Instruments, model NI USB-6211). Flow straightening grids were placed on either end of the working section. The middle of the working section was illuminated from above by light from an LED source (LED Lenser®, model P14) passed through a narrow slit (3 mm) sitting atop the working section that spanned the length of the chamber across the middle of the working section.

Larval swimming behavior was initially measured in still water in the presence of substrates containing a settlement cue. Cue-laden substrates were prepared in two ways:

1. Slide treatment: Crustose coralline algae (CCA) chips and attached coral matrix collected from the field were dried, pulverized, and then secured to a standard glass microscope slide with silicone adhesive. The slide was cured for 12 h before being placed on the floor of the flume working section.
2. Tile treatment: A rectangular fragment of a brick settlement tile (deployed onto the reef ~3 months prior and collected immediately prior to the experiment) containing live CCA and other algae (7 × 2.5 × 1 cm, L × W × H) was deposited directly onto the floor of the flume working section.

Larvae were exposed to each substrate separately and were not reused for any trials in this experiment. For each treatment, the flume was filled with FSW, and water was allowed to stabilize for ~10 min. Twelve *I. cuneata* larvae were place in the chamber with a pipette, and water motion was allowed to stabilize for 1 min. Larvae were filmed for 10 min at 30 fps across a 4 × 2 cm (W × H) FOV. Kinematic data (position, velocity, orientation, rotation) of individual larvae were tracked from recorded footage using a custom Matlab script. Footage of larvae that did not remain in the illuminated midsection of the camera’s FOV were excluded from analysis.

Flow velocities and oscillation periods for the flume experiment were set based on field flow measurements on the reef. These were calculated by manually tracking 10 particles in field PIV footage recorded near the reef crest (3 m behind ADV) and on the back reef (27 m behind ADV). Particles approximately 2 cm above the substrate were tracked using the MTtrackJ plugin^42^ in ImageJ (NIH). Near the crest, water motion oscillated between 0 and 11 cm s^−1^ while flows on the back reef oscillated between 0 and 5 cm s^−1^. These two velocity ranges simulated high-flow (crest) and low-flow (back-reef) flume conditions, with a 3 s oscillation period. Flow patterns were calibrated by manually tracking video footage of neutrally buoyant hydrated *Artemia* cysts (serving as passive particles) and adjusting the analog voltage signal to the servomotor until the velocity ranges matched the above values. Initial trials showed that larvae exposed to the high-flow regime had no chance of successful attachment. As a result, only low-flow conditions were used in all trials of this experiment.

Larvae were filmed in oscillating low-flow conditions for three surface topography treatments:

1. Slide treatment. FOV: Midsection of the glass slide, including substrate.
2. Tile treatment. FOV: Two-thirds downstream of the tile’s leading edge, to avoid capturing leading edge vortices.
3. Block treatment: A small, rectangular section (1 × 2.5 × 1 cm, L × W × H) of the same settlement tile from treatment 2 was mounted on a glass slide with silicone adhesive (Fig. 1c). FOV: The entire block surface.

The three treatments represent conditions of increasing local turbulence within the FOV as a result of increasing topographical or structural complexity, which was predicted to increase larval contact with the substratum. Before each treatment, the flume was emptied and rinsed of larvae and particles then filled with fresh FSW. For each treatment, approximately 60 *I. cuneata* larvae were introduced into the flume via pipette while water was in motion. The experimental setup was allowed to stabilize for 2 min, then the working section of the flume was filmed for ~1 h. Subsequently, ~100 neutrally buoyant *Artemia* cysts (passive particles) were then introduced by pipette and filmed for approximately 10 min to characterize flow conditions. Successful adhesion (attachment to substrate for at least 5 min after initial contact) was observed solely in the block treatment. The kinematic data of larvae were tracked using a custom Matlab script, and incidences of contact with the substrate (either followed by successful attachment or immediate detachment) were recorded.

### Analysis of larvae contact and attachment

To estimate statistical differences among topography treatments and live and dead larvae, we used bias-reduced logistic multiple regression to avoid complete separation in non-block treatments that had none or few successes^43^. Bais-reduced generalized linear models with logit link function were run for larvae contact and attachment separately using R^36^ and the *brglm* function and package^44^.

### Analysis of larval motion

Median and interquartile range swimming speeds (velocity magnitudes regardless of direction) of individual larvae in still-water conditions were determined from the instantaneous frame-by-frame velocities per individual. To measure larval swimming performance in oscillating flow, vertical velocities (w) of larvae and neutral particles were compared in the slide and tile treatments.

### Data accessibility

Larval swimming data are deposited at the Coral Trait Database^45^: https://coraltraits.org/traits/169.

## Results and Discussion

**Figure 2:**
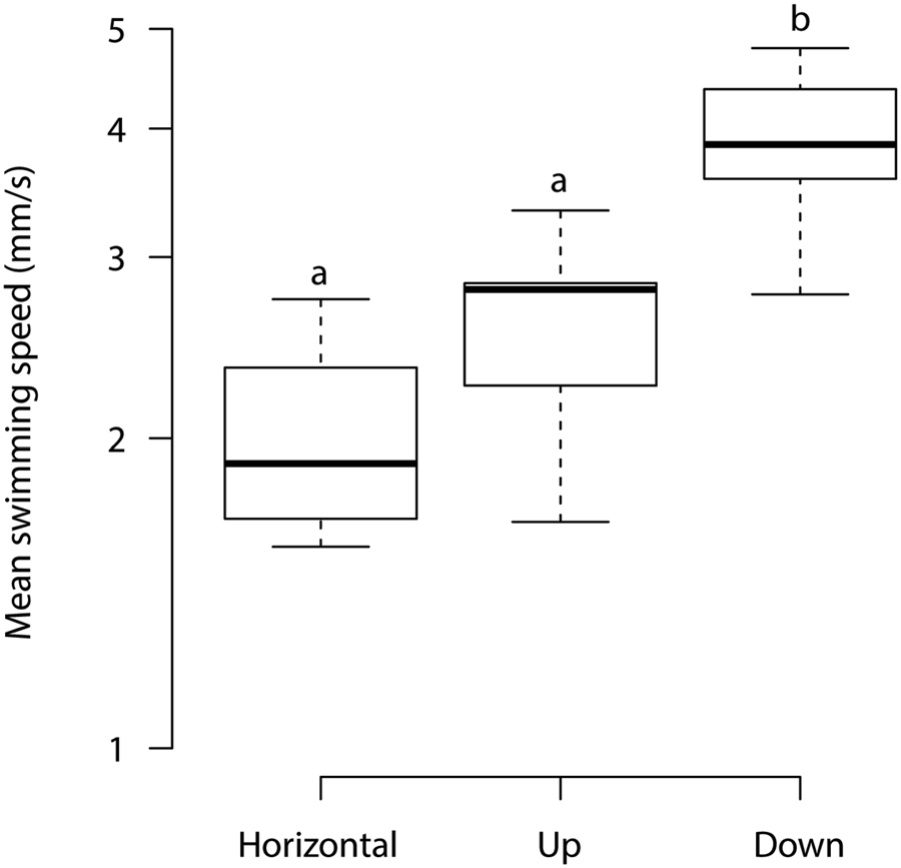
Mean larval swimming speeds in three different directions, horizontal, vertical and downward (i.e., gravity assisted), taken from nine studies representing over 450 individual measurements of swimming speeds 24. Standard errors illustrate statistical differences; the characters (**a**) and (**b)** denote significant differences at alpha = 0.05, post-hoc Tukey’s test.

Our meta-analysis of coral larval swim speeds^46–53^ (Table 1) shows that swimming speeds are much lower than the speeds of the tidal currents and orbital wave motions found in and around coral reefs (Table 2). Coral larval swimming speeds were not associated with larval size but did vary with the direction of swimming (F_2,15_ = 13.72, *p* < 0.001). Larvae swam faster when heading downwards than in the horizontal or upwards direction (Fig. 2). Mean swimming speeds for each species ranged from 1.57 mm s^−1^ for *Heliofungia actiniformis*, swimming horizontally, to 4.79 mm s^−1^ for *Pocillopora damicornis*, swimming downward (Table 1). The range of swimming speeds among all 450 measurements was 0.08 mm s^−1^ to 6.49 mm s^−1^ (Table 1). Horizontal water current speeds in the ocean were 1–4 orders of magnitude greater than larval horizontal swimming speeds, and vertical water current speeds were 1–3 orders of magnitude greater than larval vertical swimming speeds (Tables 1 and 2). In addition, periods of slack water (i.e., the time when current speeds are potentially slow enough to allow larvae to make headway; less than 5 mm s^−1^ based on maximum swimming speeds in Table 1) were extremely limited over this two-week period (Table 2). Horizontal current speeds never dropped below this 5 mm s^−1^ threshold, and vertical currents were greater than the threshold for over 90% of the time (Table 2). We conclude that coral larval swimming speeds^20^ are orders of magnitude lower than measurements of water flow both on and off reefs. Therefore, the ability of coral larvae to navigate to reefs while in the open-ocean, or to settlement sites while on reefs is extremely limited. Even if coral larvae can distinguish between waters from healthy and degraded reefs^33^, they will not be able to navigate to healthy reefs because their swimming speeds are far too low to overcome currents.

**Table 2:** Minimum and maximum observed current speeds (left-hand panes) during the 2 weeks following coral mass spawning in 6 successive years at 3 locations in the Great Barrier Reef, and the total length (and percentage) of time during this two-week period when current speeds were under 5 mm s^−1^ (right-hand panes).

Year	Lizard Island Shelf	Heron Island North	Heron Island South	Lizard Island Shelf	Heron Island North	Heron Island South
	**Depth-average of horizontal current speeds (min – max, mm s** ^**−1**^ **)**			**Total period with horizontal current speeds below 5 mm s** ^**−1**^ **(min)**		
2007	n/a	133–946	54–1097	n/a	0	0
2008	52–1096	175–1016	104–979	0	0	0
2009	n/a	n/a	80–1049	n/a	n/a	0
2010	104–1170	118–1364	122–1378	0	0	0
2011	n/a	123–1864	70–1292	n/a	0	0
2012	24–1348	n/a	16–1410	0	n/a	0
	**Depth-average of vertical current speeds (min – max, mm s** ^**−1**^ **)**			**Total period with vertical current speeds below 5 mm s** ^**−1**^ **(min)**		
2007	n/a	2–77	4–240	n/a	1710 (8.4%)	180 (0.9%)
2008	1–22	4–109	6–72	11820 (58.5%)	90 (0.5%)	0
2009	n/a	n/a	3–143	n/a	n/a	150 (0.7%)
2010	19–135	5–297	3–164	0	10 (0.1%)	60 (0.3%)
2011	n/a	2 – 424	3–163	n/a	10 (0.1%)	210 (1.0%)
2012	2–132	n/a	2–238	1000 (5%)	n/a	160 (0.8%)

The speeds shown are averages of the speeds of horizontal (upper table) and vertical (lower table) currents over the top 20 m of the water column, and were obtained from the Great Barrier Reef Ocean Observing System^23^. Years for which data were not available are marked as n/a.

**Figure 3:**
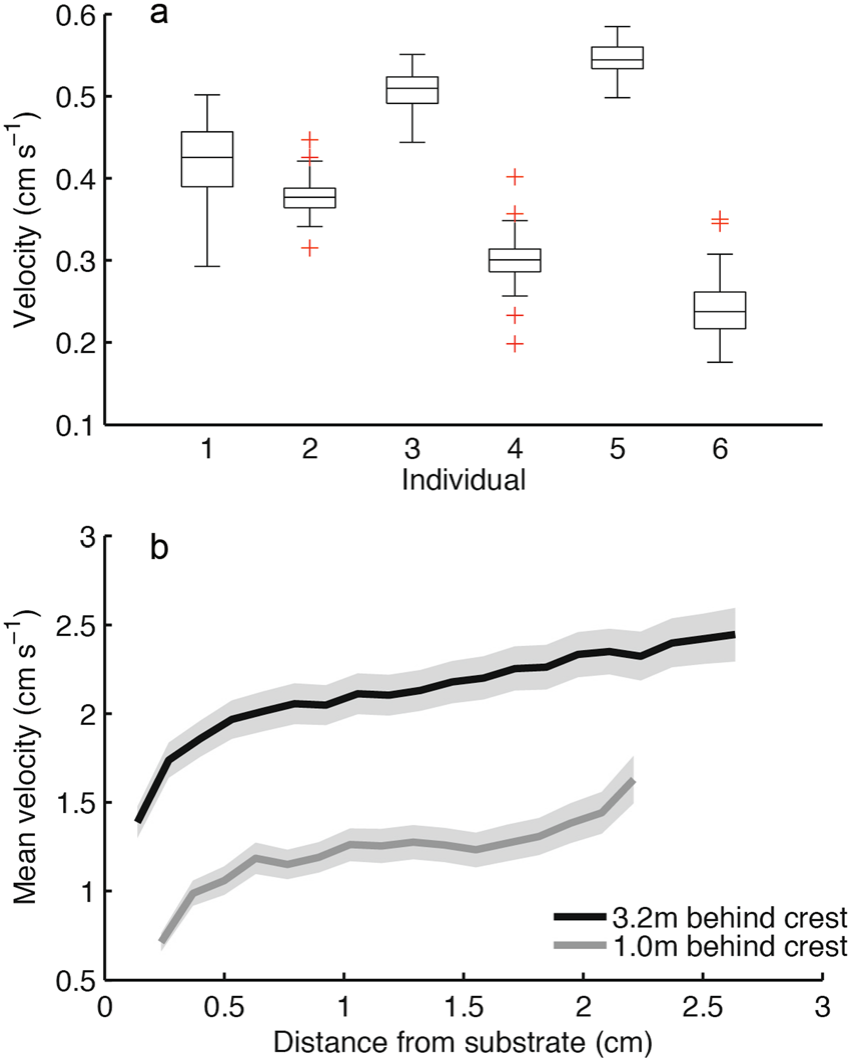
Swimming on the reef. (**a**) Swimming speeds of *Isopora cuneata* larvae measured in still water. Box plots show the median, interquartile range, ±1.5 interquartile range with outliers in red. (**b**) Mean water velocities above the substratum at two positions back from the reef edge at Lizard Island (Great Barrier Reef, Australia) measured using particle image velocimetry. Grey bands represent 99% confidence intervals.

**Figure 4:**
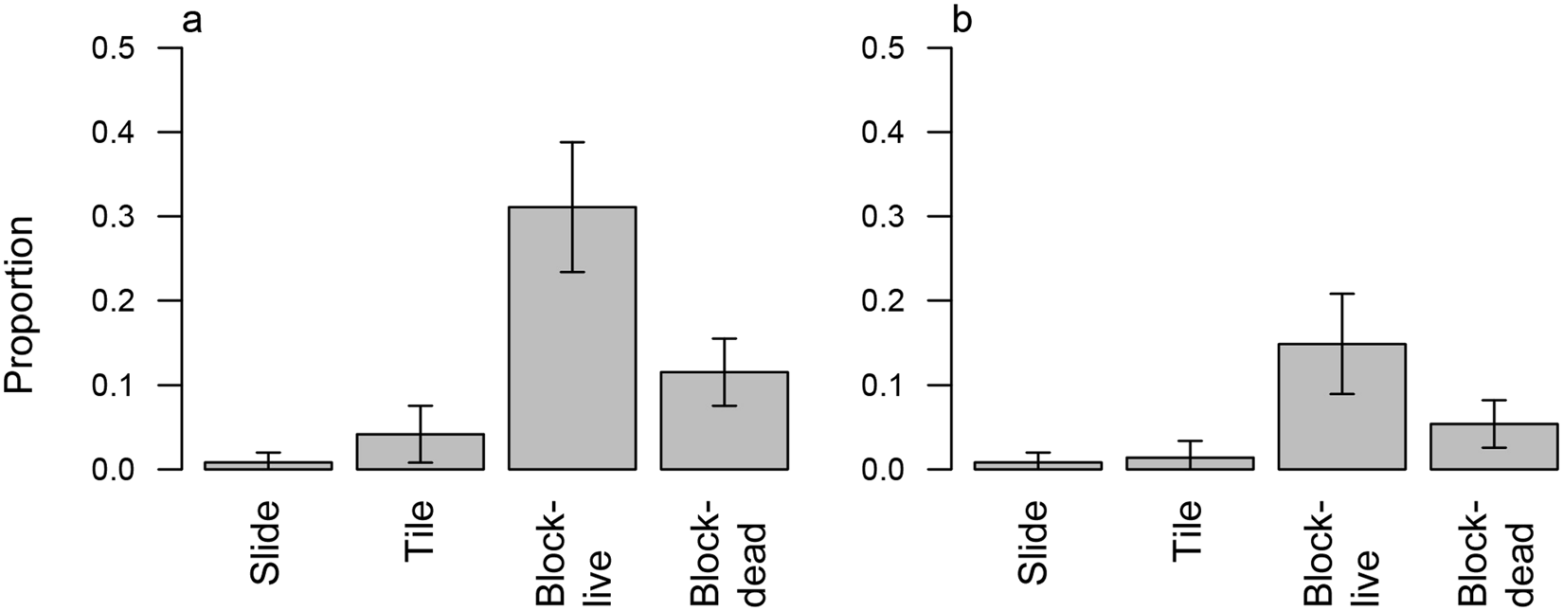
Settlement success of coral larvae in oscillating flume tank. Proportion of *Isopora cuneata* larvae making (**a**) contact with settlement substrates covered with CCA and forming (**b**) attachment for more than 5 min. Substrate treatments were: microscope slide, flat settlement tile and block. For substrates where contact was observed, flume experiments were run separately with live and dead larvae. Standard errors illustrate statistical differences; also see Table 3.

Our meta-analysis indicated that the genus *Isopora* contains particularly fast-swimming larvae (Table 1) and this was confirmed in the flume where *I. cuneata* larvae ranked among the fastest larvae, with swim speeds up to 5.8 mm s^−1^ (Fig. 3). However, this high swimming capacity did not translate into a high capacity for settlement. Of the 95 *I. cuneata* larvae tracked in the flume, only one made contact with the substratum in the tile treatment and none became attached (Fig. 4). Attachment only occurred when a protruding structure in the form of a block was introduced to break up the flow (Fig. 4b). For the block treatment, contact and attachment also occurred regardless of whether larvae were dead or alive (Fig. 4) although both contact and attachment were higher for live larvae (Table 3). Indeed, our estimates of current velocities on the reef substratum (Fig. 3b) suggest that turbulence generated by the boundary layer^19^ is insufficient to enable coral larvae to settle. We suggest that without the additional turbulence and eddies generated by complex micro-structure coral larvae will not be able to navigate to the substratum even when exposed to low-flow, back-reef flow conditions. We conclude that this fine-scale structure assists coral recovery from disturbance by facilitating both the delivery of coral larvae to the substratum and settlement. We further hypothesize that structural complexity at a larger scale^4^ works in a similar way by creating turbulence to capture larvae from the water column as they flow across the reefs.

**Table 3:** Summaries of larvae contact (upper table) and attachment (lower table) bias-reduced logistic regression models.

	Estimate	Std. Error	z value	Pr(>|z|)
**Contact**				
(Block, Dead)	−2.0369	0.3913	−5.206	0.000
*Slide*	−*3.9995*	*1.4764*	−*2.709*	*0.007*
Tile	−2.3392	0.9194	−2.544	0.011
Live	1.2406	0.5317	2.333	0.020
**Attachment**				
(Block, Dead)	−2.8663	0.5538	−5.176	0.000
*Slide*	−*3.0506*	*1.5065*	−*2.025*	*0.043*
Tile	−2.5174	1.5184	−1.658	0.097
Live	1.1210	0.7254	1.545	0.122

In conclusion, despite the well-documented sensory capacity of coral larvae^16, 17^, their swimming speeds are much too low to enable them to navigate to suitable settlement sites under most conditions. When in the open ocean, and even on reefs, coral larvae are essentially lost at sea^10^. Furthermore, because flows that are sufficiently benign for coral larvae to swim directly to the substratum are very rare, some form of topographic structure is required to generate turbulence to capture larvae from the plankton and to deliver them to the substratum. We suggest that this is one mechanism by which structural complexity promotes reef recovery following disturbance^4^. Consequently, maintaining structural complexity at a number of scales on reefs is vitally important in terms of aiding reef recovery. Relevant management actions include limiting factors that reduce complexity, such as destructive fishing practices^54^ and promoting factors that enhance complexity, such as herbivory^11, 12^.
